# Asymmetric volatility in asset prices: An explanation with mental framing

**DOI:** 10.1016/j.heliyon.2024.e24978

**Published:** 2024-01-19

**Authors:** Mihály Ormos, Dusán Timotity

**Affiliations:** a Department of Economics, J. Selye University, Hradná ul. 21., 94501 Komárno, Slovakia; b Department of Finance, Budapest University of Technology and Economics, Magyar tudosok krt. 2., 1117 Budapest, Hungary

**Keywords:** Asymmetric volatility, Mental framing, Volatility dynamics, Market microstructure, Heuristic-driven trader

## Abstract

We propose a theoretical framework for the heteroscedasticity, and in particular for the asymmetric volatility of asset returns. Our model is based on the assumption that some investors are subject to mental framing in a dynamic setting. The analysis of individual trading data confirms that, in line with our model, investors tilt their portfolio towards riskier (less risky) assets subsequent to losses (gains). Based on their behavior, we derive a volatility process that accounts for the asymmetry thoroughly investigated in previous empirical studies: the parameter estimation of our volatility model yields the predicted negative relationship between abnormal returns and ensuing volatility.

## 1 Introduction

Modelling asset price fluctuations has always been a main area of research since the appearance of financial theory. In particular, the amplitude at which prices fluctuate, mainly measured by the standard deviation of returns (also known as volatility) plays a crucial role in risk-management and portfolio theory, two prominent fields in finance. Therefore, understanding the structure of the long-established dependency of volatility on its past and its relationship with asset returns still attracts a wide researcher community both from academy and practice alike. In contrast to the extensive amount of findings in the topic, the path-dependency of volatility and in particular, its negative relationship with asset returns (also called asymmetric volatility) still remain to be explained in a fundamental way. In this paper, we aim to bridge this gap by connecting a pattern in investor behavior (mental framing) with empirical findings on portfolio choice and asset price fluctuations.

### 1.1 Literature on volatility

In recent decades, time-varying volatility (heteroscedasticity) of asset returns has attracted much research, especially since the milestone papers of [1] Engle (1982) and [2] Bollerslev (1986), whose findings indicate that the dynamics can be modeled by generalized autoregressive conditional heteroscedasticity (GARCH) type models. Although, heterogenous agent-based models, including behavioral financial findings ([3] Lux and Marchesi, 2000 [4]; Shimokawa et al., 2007), have provided simulation results supporting this latter phenomenon, a general analytical solution for the existence of volatility persistence has not been derived from the perspective of investors’ portfolio allocation.

In addition to the relationship with their past, asset prices show a surprising pattern that is yet to be explained: the asymmetry in their volatility process. In particular, “asymmetric volatility” implies that changes in the price of the underlying asset are negatively correlated with the volatility of the subsequent period. Despite the copious literature devoted to asymmetric volatility ([5] Black, 1976 [6]; Christie, 1982; and [7] Schwert, 1989), a robust explanation for this phenomenon is still lacking.

In this paper, we propose a theoretical explanation supported by empirical evidence for the existence of such an autoregressive and asymmetric volatility process in a framework based on intertemporal mental framing. First, by using a unique dataset that contains individual investors' trades for a five-year period ([8] Barber and Odean, 2000), we show that investors are subject to mental framing, that is, they aggregate their required return in time, previous shocks hence affect their portfolio choice: portfolio shocks are negatively correlated with the required return of the subsequent period. We measure that their mental framing leads to excess demand (supply) of risky assets subsequent to negative (positive) market shocks. Second, we present a market microstructural channel through which this heuristic-driven demand affects the equilibrium bid-ask spread, and therefore drives the volatility process. Hence, altogether, our findings reveal a framework, in which previous market jumps (falls) increase (decrease) subsequent market-wide and asset-specific volatilities, providing an explanation for asymmetric volatility.

### 1.2 Volatility and expected return

The analysis of the relationship between volatility and expected returns dates back to the very beginning of modern finance. The principle of a positive risk-return relationship has been documented in thousands of research papers for a large number of risk measures. In this current paper, as we assume market frictions (e.g. the imperfect diversification of investors’ portfolio and non-zero bid-ask spreads), we chose an asset-specific measure as the proxy for risk, the idiosyncratic volatility (i.e. the riskiness of individual asset returns unrelated to the market movements).

According to Ref. [9] Merton (1987), due to under-diversification and market frictions, this measure drives asset prices, which is reflected by a positive relationship between risk and return. This relationship between idiosyncratic risk and expected return has been confirmed empirically using diverse settings ([10] Bali and Cakici, 2008 [11]; Malkiel and Xu, 2002). Although, some authors have found that similar risk measures, such as the sensitivity to market-wide risk ([12] Ang et al., 2006), have a negative effect on the expected return, controlling for lagged returns and volatilities [13] Fu (2009) has resolved this anomaly, and provided further evidence for the positive relationship. These latter results have highlighted that lagged terms of volatility and return are relevant in determining asset price dynamics. In this paper, therefore, we include the effects of both idiosyncratic and market-wide volatilities and also their lagged values for determining the relationship between risk and reward.

However, since we focus on defining the volatility dynamics instead of predicting future returns, market and asset-specific returns and their lagged values are the independent variables.

This kind of reverse-order analysis is not new in related research; in particular, volatility forecasting and analysis rely on the persistence of risk and volatility clustering. In addition to the persistence, researchers have found an asymmetric effect of preceding shocks on the dynamics of volatility. This phenomenon is called asymmetric volatility meaning that previous positive and negative shock have significantly different effect on volatility.

Until now, three main explanations have been proposed for the asymmetric volatility puzzle. The first is the leverage effect noted by Refs. [[5], [6], [7]] Black (1976), Christie (1982), and Schwert (1989). The authors assume that if the value of an equity drops, the firm becomes more leveraged, therefore, the volatility of equity returns rises according to the increased risk, hence causing the negative relationship between return, and subsequent volatility. They conclude, however, that, although volatility is indeed an increasing function of financial leverage, the effect by itself is not sufficient to account for the observed negative correlation.

The second explanation, labeled as the volatility feedback hypothesis, states that in cases of unexpected increase in volatility (e.g. exogenous shocks), expected volatility rises accordingly, and thus increasing the required return of the given asset in line with equilibrium asset pricing models. This has an immediate negative impact on current stock price; hence, it strengthens or weakens the magnitude of a previous shock subsequent to losses or gains respectively, thereby causing the asymmetry. Numerous papers on the topic have provided evidence supporting both explanations ([14] Pindyck, 1984; or [15] Kim et al., 2004), yet recent studies still yield controversial results: on the one hand [16], Bollerslev et al. (2006) find that the analysis of high-frequency data indicates no significant volatility feedback; while on the other hand [17], Bekaert and Wu (2000) conclude that the leverage effect is insignificant.

The third main explanation is given by Ref. [18] McQueen and Vorkink (2004), proposing that volatility autocorrelation is due to investors' inclusion of the fluctuation of prices in their perceived utility (i.e. loss-averse behavior ([19] Kahneman and Tversky, 1979)). The authors assume that volatility increases both following gains and losses as in volatility feedback hypothesis. Their assumption comes from the paper of [20] Barberis, Huang and Santos (2001) (henceforth *BHS*), which provides an asset pricing interpretation of [21] Thaler and Johnson's (1990) experiment of prospect theory in a dynamic setting. BHS assume that perception of losses (or gains) is more (or less) painful (or delightful) when they follow prior losses (or gains). This means that previous losses increase and previous gains decrease risk-aversion. However, BHS do not take into account the entire analysis of [21] Thaler and Johnson; the authors do not focus on the finding that investors become risk-seeking following losses and risk-averse subsequent to gains, if the opportunity of breaking-even is included in the choice set, which, in fact, almost always applies to asset returns.

Our main contribution to related literature lies in pointing out the effects on portfolio choice and volatility of this latter phenomenon that involves intertemporal mental framing. As we show below, instead of the 10.13039/100010227BHS assumption, our individual dataset provides support for this latter risk-modifying behavior. In other words, the patterns obtained in our empirical tests suggest that, in contrast to BHS (2001) and [18] McQueen and Vorkink (2004), portfolio volatility does indeed increase after losses, and decreases subsequent to gains in order to allow, or prevent, breaking-even respectively.

The remaining part of the paper is structured as follows: in Section 2 the theoretical model is presented; Section 3 discusses the behavioral patterns emerging in the investors’ trading dataset, as well as an empirical parameter estimation of the model through a time-series analysis of asset prices. Finally, Section 4 summarizes the main conclusions of the paper and provides potential ways for further research.

## 2 The model

We find that previous market return plays a dominant role in the volatility dynamics of assets; as shown in Appendix A, it is mainly responsible for the asymmetric response of idiosyncratic volatility to shocks, leaving the effect of previous asset-specific shocks marginal. We argue that this phenomenon can be explained by applying mental framing in a dynamic setting. In the followings, we discuss our setting in detail by first defining the temporal dynamics of investors' required return, then describing the risk-return relationship, deriving the dynamics of the volatility of investors’ portfolios, and finally we give an explanation of the aggregate market volatility.

### 2.1 Dynamics of the required return

Assuming that investors hold portfolios similar to the market portfolio, or at least that they diversify and hence invest in multiple assets, the return on their portfolio should be highly correlated with the market return. Thus, a negative or positive market shock would lead to losses and gains on investors’ portfolios [21]. Thaler and Johnson (1990) show in their experimental study that in such cases (if breaking even is in the choice set), investors become risk-seeking following losses and more risk-averse subsequent to gains; they aim to avoid realizing losses (exactly as in disposition effect ([22] Shefrin and Statman, 1985, and [23] Odean, 1998)) and are afraid of losing previous paper gains. This behavior may come from the S-shaped value function of loss-aversion: if we include the previous outcome as a reference point, the convexity of utility perception in the domain of losses results in risk-seeking behavior as the expected utility reaches its maximum at positive risk. In this specific case, and considering the previous outcome as a fixed loss, it causes greater pain than aggregating over time and hoping to break even; however, realizing the previous gain yields higher expected utility than taking the risk of losing the accumulated wealth.

Therefore, mental framing (the mental aggregation or separation of pieces of information) in a dynamic setting leads investors to aggregate in time. Hence, they aim to obtain a given reference return at each period or, at least, earn this return on average. That is, if we assume the rational expectations of outcomes as the reference point ([24] Koszegi and Rabin, 2006), the subsequent required return is decreased by the previous abnormal return to be able to obtain the pre-defined reference return on average.

Analytically, let $\mu_{t}$ define investors’ required return in period *t*. Assuming that they allocate their portfolios rationally, this is equal to the expected return. Since they had an expectation in the past also, their previous abnormal gain or loss was $r_{t-1}-\mu_{t-1}$. As mentioned above, mentally realizing this loss (or gain) causes a greater pain (or higher utility) than accepting a risky investment; therefore, a negative (or positive) $r_{t-1}-\mu_{t-1}$ shock increases (or decreases) the subsequent required, and expected, return by a magnitude of α, which stands for the negative relationship between shock and subsequent required return. Then, the aforementioned behavior can be described with equation (1).

(1) $$\mu_{t}=\mu_{t-1}+\alpha \left(r_{t-1}-\mu_{t-1}\right)+r_{f,t}-r_{f,t-1}:\alpha \in \left[-1,0\right]\text{,}$$

where $r_{f,t}$, $r_{t}$ and $\mu_{t}$ stand for the risk-free rate, the portfolio return, and the required/expected portfolio return of a given investor respectively. Here, the economic interpretation of α is defined as the sensitivity of an investor to mental framing (the aggregation of previous outcomes). It makes no sense to assume that market participants adjust the required return by more than the previous shock itself; hence, we set its lower boundary at −1. Its negative sign is due to the definition: aggregating over time increases or decreases the required return subsequent to losses or gains respectively. The $r_{f,t}-r_{f,t-1}$ terms are added as the correction for the change in the risk-free rate, or inflation. We emphasize that evidence for the aforementioned patterns in the dynamics of risk-seeking, reference-dependence, and framing have also been bolstered by numerous studies from alternative fields of science such as neuroeconomics ([25] Kuhnen and Knutson, 2005), and non-human physiology ([26] Chen et al., 2006 [27]; Lakshminarayanan et al., 2011).

### 2.2 The dynamics of portfolio volatility

We define the intertemporal change of volatility in Eqs. (2), (3); we assume that (i) in line with [9] Merton (1987), expected returns are driven by total volatility $\sigma_{t}$ in a market with frictions: in particular, the risk premium is a linear function of risk with a slope β; (ii) the price of risk β does not change over time. In this setting, the required return is not constant but follows the dynamics of

(2) $$\mu_{t}=r_{f,t}+\beta \sigma_{t}=\mu_{t-1}+\alpha \left(r_{t-1}-\mu_{t-1}\right)+r_{f,t}-r_{f,t-1}==r_{f,t}+\beta \sigma_{t-1}+\alpha \left(r_{t-1}-r_{f,t-1}-\beta \sigma_{t-1}\right)\text{.}$$

The economic interpretation of Eq. (2) is that subsequent to losses, investors aim to obtain a higher expected return; however, according to equilibrium pricing, they can only achieve their goal by investing in riskier assets or increasing leverage. Solving the latter equation for the dynamics of volatility yields

(3) $$\sigma_{t}=\sigma_{t-1}+\frac{\alpha }{\beta }\left(r_{t-1}-r_{f,t-1}-\beta \sigma_{t-1}\right)=\sigma_{t-1}+\frac{\alpha }{\beta }e_{t-1}==\sigma_{t-1}+\frac{\alpha }{\beta }\sigma_{t-1}{\Delta W}_{t-1}=\sigma_{t-1}\left(1+\frac{\alpha }{\beta }{\Delta W}_{t-1}\right)\text{,}$$

where $e_{t-1}$ and ${\Delta W}_{t-1}$ represent an error term and the change in the standard Wiener process in discrete time if standard normal distribution is assumed for security returns. Here we note that the error term may be generalized to any distribution such as in Refs. [28,29] Schoutens (2001, 2003) [30], Stein and Stein (1991) or [31] Carr and Wu (2004); however, in order to create the least complex model based on only the very fundamental properties of human behavior and the Central Limit Theorem, we assume normality at this point.

Eq. (3) reveals that $\sigma_{t}$ follows a unit-root process with constant conditional mean, that is

(4) $$E\left[\sigma_{t+\tau }|F_{t}\right]=\sigma_{t}+E\left[\sum_{i=t}^{t+\tau -1}\frac{\alpha }{\beta }\sigma_{i}{\Delta W}_{i}|F_{t}\right]=\sigma_{t}+\frac{\alpha }{\beta }\sum_{i=t}^{t+\tau -1}E\left[\sigma_{i}|F_{t}\right]E\left[{\Delta W}_{i}|F_{t}\right]=\sigma_{t}+\frac{\alpha }{\beta }{\Delta W}_{t}$$

where $F_{t}$ stands for the filtration (information available) at time *t*. Here, the separation of contemporaneous volatility and noise requires the assumption that they are uncorrelated (only the delayed response yields a negative correlation). According to Eq. (4), the volatility process seems to be valid and realistic, in the sense that periodical volatility tends to remain in a finite interval over a long horizon: it converges neither to infinity, nor to zero. Furthermore, Eq. (3) reveals another interesting pattern: it is very similar to the Treshold GARCH (TGARCH) model introduced by Ref. [32] Zakoian (1994) (or GJR-GARCH [33] (1993) in a non-squared setting) – one of the most accurate heteroscedasticity models based on goodness-of-fit tests ([34] Awartani and Corradi, 2005 [35]; Tavares et al., 2008). In particular, TGARCH models are defined as per Eq. (5) of

(5) $$\sigma_{t}=K+\delta \sigma_{t-1}+\alpha^{+}{e_{t-1}}^{+}+\alpha^{-}{e_{t-1}}^{-}$$

where ${e_{t-1}}^{+}=\left\{ \begin{array}{c} e_{t-1}\;if\;e_{t-1}>0\\ 0\;if\;e_{t-1}\leq 0 \end{array}\right.$ and ${e_{t-1}}^{-}=\left\{ \begin{array}{c} e_{t-1}\;if\;e_{t-1}\leq 0\\ 0\;if\;e_{t-1}>0 \end{array}\right.$. Therefore, the special case of Eq. (3) implies that K=0, δ=1 and $\alpha^{+}=\alpha^{-}=\frac{\alpha }{\beta }$.

Effects of previous gains and losses could also be handled separately in Eq. (3) by using different $\alpha^{+}$ and $\alpha^{-}$, since as we show below, previous gains only play a much less significant role in the asymmetric effect on volatility. Moreover, distinct $\alpha^{+}$ and $\alpha^{-}$ would also have a reasonable economic interpretation: considering that extreme gains do not cause a negative required return, that is, investors cannot, and will not, invest in assets with negative expected return irrespective of the previous outcomes, gains should have a less significant effect on the subsequently required return; therefore, $\alpha^{+}$ should differ from $\alpha^{-}$, which causes further asymmetry in addition to the different effect of previous gains and losses.

This combination of unit-root volatility with asymmetric effect to previous shocks is consistent with various empirical findings. In particular, the random walk model is already found to perform very well in some markets ([36] McMillan et al., 2000); however, the integrated, and especially, the asymmetric and fractionally integrated GARCH models ([37] Baillie et al., 1996) are commonly documented to outperform their alternatives ([38] Conrad et al., 2011 [39]; Kilic, 2011). These could be an extension of our proposed model, assuming that investors include outcomes beyond the first lag in allocation decisions.

Another interpretation of Eq. (3) leads to a further well-fitting, asymmetric GARCH model: the Exponential Generalized Autoregressive Conditional Heteroscedasticity (EGARCH) by Ref. [40] Nelson, (1991). Dividing by $\sigma_{t-1}$ and taking the natural logarithms of both sides yields

(6) $$\ln\;\sigma_{t}=\ln\;\sigma_{t-1}+\ln\left(1+\frac{\alpha }{\beta }{\Delta W}_{t-1}\right)\text{.}$$

Taking the Taylor approximation around ${\Delta W}_{t-1}=0$ then gives Eq. (7) as

(7) $$\ln\;\sigma_{t}=\ln\;\sigma_{t-1}+\frac{\alpha }{\beta }{\Delta W}_{t-1}-\frac{1}{2}{\left(\frac{\alpha }{\beta }\right)}^{2}{{\Delta W}_{t-1}}^{2}+\sum_{n=3}^{\infty }\frac{{\left(-1\right)}^{n-1}}{n!}{\left(\frac{\alpha }{\beta }\right)}^{n}{{\Delta W}_{t-1}}^{n}\text{.}$$

Due to the well-known property of the Wiener process, as *Δt* tends to zero (the continuous time version is considered) third and higher order polynomials of ${\Delta W}_{t}$ vanish and ${{\Delta W}_{t}}^{2}=\Delta t$. Therefore, Eq. (6) in continuous time can be written as Eq. (8).

(8) $$\ln\;\sigma_{t}=\ln\;\sigma_{t-1}+\frac{\alpha }{\beta }{dW}_{t-1}-\frac{1}{2}{\left(\frac{\alpha }{\beta }\right)}^{2}dt\text{,}$$

or by multiplying both sides by 2 yields Eq. (9) as

(9) $$\ln\;{\sigma_{t}}^{2}=\ln\;{\sigma_{t-1}}^{2}+2\frac{\alpha }{\beta }{dW}_{t-1}-{\left(\frac{\alpha }{\beta }\right)}^{2}dt\text{.}$$

The similarity to EGARCH comes from its definition of Eq. (10) as

(10) $$\ln\;{\sigma_{t}}^{2}=\omega +\beta_{1}\left[\theta {dW}_{t-1}+\lambda \left(\left|{dW}_{t-1}\right|-E\left|{dW}_{t-1}\right|\right)\right]+\alpha_{1}\;\ln\;{\sigma_{t-1}}^{2}\text{,}$$

where $\omega =-{\left(\frac{\alpha }{\beta }\right)}^{2}dt$, $2\frac{\alpha }{\beta }=\beta_{1}\theta$, λ=0 and $\alpha_{1}=\alpha$ yields exactly Eq. (10). The unit-root, constant conditional mean property of Eq. (9) is again found by applying Itō’s lemma for Eq. (11)

(11) $$x_{t}\equiv \ln\;{\sigma_{t}}^{2},dx_{t}=2\frac{\alpha }{\beta }{dW}_{t}-{\left(\frac{\alpha }{\beta }\right)}^{2}dt\text{.}$$

Then the inverse function is defined as Eq. (12).

(12) $$\sigma_{t}=e^{0.5x_{t}}\text{.}$$

By Itō’s lemma we get Eq. (13) as

(13) $$d\sigma_{t}=\left[-{\left(\frac{\alpha }{\beta }\right)}^{2}\frac{\partial \sigma_{t}}{\partial x_{t}}+\frac{1}{2}{\left(2\frac{\alpha }{\beta }\right)}^{2}\frac{\partial^{2}\sigma_{t}}{\partial {x_{t}}^{2}}\right]+2\frac{\alpha }{\beta }\frac{\partial \sigma_{t}}{\partial x_{t}}{dW}_{t}==\left[-{\left(\frac{\alpha }{\beta }\right)}^{2}0.5\sigma_{t}+\frac{1}{2}{\left(2\frac{\alpha }{\beta }\right)}^{2}0.25\sigma_{t}\right]+2\frac{\alpha }{\beta }0.5\sigma_{t}{dW}_{t-1}=\frac{\alpha }{\beta }\sigma_{t}{dW}_{t}\text{.}$$

Thus, the correlation between concurrent volatility and noise has zero expected value, therefore, the conditional mean is constant regardless of the length of delay. To conclude, the TGARCH and EGARCH models for portfolio volatility are implications of mental framing in a dynamic setting and they represent the underlying volatility process in discrete and continuous time respectively.

Modelling volatility and asset prices with similar dynamics based on Prospect Theory have already been considered in existing literature; see Rachev et al., 2017 [41] and Shirvani, 2021 [42] where the authors present how to reconcile such behavioral financial findings with rational option pricing.

### 2.3 The dynamics of asset price volatility

We have, so far, derived the change of investors' risk attitude and the dynamics of the volatility of their portfolios. However, increased leverage or tilt towards riskier assets may induce higher portfolio volatility for investors without affecting market-wide uncertainty (e.g. even if every investor held the market portfolio they could all increase their volatility using higher leverage without affecting the volatility present in a frictionless market); hence, reasons behind the change of asset volatility have not yet been covered. In this section, we propose an explanation for the positive relationship between the dynamics of price volatility and the riskiness of investors’ portfolios based on a simple market microstructural idea.

As discussed above, mental framing leads to a clear pattern in investor's choice that depends on the previous unexpected price shock: losses increase the subsequent demand for risky assets, whereas, gains reduce their demand. If we stick to the idea that – as assumed in Section 2.2 –, investors hold the market portfolio, or at least a mixture of assets that is correlated with the market, one can clearly see the following market microstructural situation: in line with the model of [43] Glosten and Milgrom (1985), we find informed and uninformed traders in the market with probabilities π and (1−π) who place market orders. In their model, the informed investors know the exact value of an asset that can be either high ($v^{H}$), or low ($v^{L}$) and place their orders accordingly. Other participants of the market, such as the specialists who provide liquidity by placing limit orders (thus define the spread) know only the probability of the true value: $P\left(v=v^{H}\right)=\theta$ and $P\left(v=v^{L}\right)=1-\theta$. Uninformed investors place their buy and sell orders randomly; hence, the probabilities of buy and sell orders coming from uninformed traders are equal (P=0.5).

Therefore, the profit of specialists is generated by the losses on transactions with informed investors and gains on transactions with uninformed investors. If we assume the market is competitive, their zero expected profit criteria for transactions at the buy limit price and at the sell limit price (i.e. they lose the same expected amount of profit on informed traders with probability π as they win on the uninformed with probability (1−π)) yield the equilibrium ask and bid prices respectively (with the spread as their difference).

However, if we introduce the pattern discussed in the previous sections, the spread changes in the following way: let us assume that, based on the mental framing heuristic, there is a new type of investor in addition to informed and uninformed traders – the heuristic-driven investor. This latter definition is not new in related literature; although, according to the pioneering papers of [43] Glosten and Milgrom (1985), and [44] Kyle (1985), uninformed traders are defined as those who do not possess fundamental information on assets, irrespective of their motives, a definition similar to our setting has already appeared in the paper of [45] Bloomfield et al. (2009b), in which uninformed investors can have other trading motives than fundamental (e.g. behavioral). In their study the similar three-class distinction of investors is analyzed, where informed and uninformed investors, and liquidity traders are present. The liquidity trader, however, may follow a behavioral pattern according to the dynamics of liquidity demand we have discussed so far; hence, we call this class the heuristic-driven trader.

Turning back to the introduction of such traders in the equilibrium criteria, let π and δ and (1−π−δ) stand for the shares of informed, heuristic-driven and uninformed traders (the probability of their trades). In line with the relevant literature, we assume that all the above classes of investors trade with market orders and market makers set the bid-ask spreads with the knowing the underlying microstructure (i.e. the participation ratio of each class in orders) during all times; we further assume a competitive market for the latter, hence zero expected profit from all trades aggregated ([43] Glosten and Milgrom (1985)). Then, subsequent to a negative market shock, the zero profit criteria of specialists at the ask and bid prices can be defined as Eq. (14) and Eq. (15).

(14) $$\theta \pi \left(a-v^{H}\right)+\delta \left(a-v\right)+0.5\left(1-\pi -\delta \right)\left(a-v\right)=0\text{,}$$

(15) $$\left(1-\theta \right)\pi \left(\;v^{L}-b\right)+0.5\left(1-\pi -\delta \right)\left(v-b\right)=0\text{,}$$

Then the ask price is given as Eq. (16).

(16) $$\frac{\theta \pi v^{H}+0.5\left(1-\pi +\delta \right)v}{\theta \pi +0.5\left(1-\pi +\delta \right)}=v+\frac{\theta \pi \left(v^{H}-v\right)}{\theta \pi +0.5\left(1-\pi +\delta \right)}=v+\frac{\theta \pi \left(1-\theta \right)\left(v^{H}-v^{L}\right)}{\theta \pi +0.5\left(1-\pi +\delta \right)}$$

whereas the bid price follows as Eq. (17)

(17) $$\frac{\left(1-\theta \right)\pi v^{L}+0.5\left(1-\pi -\delta \right)v}{\left(1-\theta \right)\pi +0.5\left(1-\pi -\delta \right)}=v+\frac{\left(1-\theta \right)\pi \left(v^{L}-v\right)}{\left(1-\theta \right)\pi +0.5\left(1-\pi -\delta \right)}=v-\frac{\theta \pi \left(1-\theta \right)\left(v^{H}-v^{L}\right)}{\left(1-\theta \right)\pi +0.5\left(1-\pi -\delta \right)}$$

One can recognise the underlying economic processes in the aforementioned formulas: if heuristic-driven traders are present; the mid-price differs from the expected value. Subsequent to a negative shock, the δ proportion of investors place buy orders at the ask price; however, they do not form supply at the bid price. Furthermore, their uninformed trades contribute positively to the profit; therefore, the equilibrium ask price declines as in Eq. (16). Still, their existence decreases the proportion of uninformed investors; hence, the equilibrium bid price also declines as in Eq. (17). Although, both the ask and bid prices decline, the zero profit remains intact due to the modified probabilities of incoming buy and sell orders.

Then, the spread in competitive equilibrium can be defined as

(18) $$S_{-}=\frac{\theta \pi \left(1-\theta \right)\left(v^{H}-v^{L}\right)}{\theta \pi +0.5\left(1-\pi +\delta \right)}+\frac{\theta \pi \left(1-\theta \right)\left(v^{H}-v^{L}\right)}{\left(1-\theta \right)\pi +0.5\left(1-\pi -\delta \right)}=\frac{\theta \pi \left(1-\theta \right)\left(v^{H}-v^{L}\right)}{\left[\theta \pi +0.5\left(1-\pi +\delta \right)\right]\left[\left(1-\theta \right)\pi +0.5\left(1-\pi -\delta \right)\right]}$$

where $S_{-}$ stands for the spread subsequent to a negative market shock. The spread following positive market shocks is similar except for the sign of δ in the form of Eq. (19):

(19) $$S_{+}=\frac{\theta \pi \left(1-\theta \right)\left(v^{H}-v^{L}\right)}{\left[\theta \pi +0.5\left(1-\pi -\delta \right)\right]\left[\left(1-\theta \right)\pi +0.5\left(1-\pi +\delta \right)\right]}\text{.}$$

Let the spread be defined as a function of Δ where

$$\Delta =\left\{ \begin{array}{c} \delta \;for\;negative\;previous\;shocks\\ -\delta \;for\;positive\;previous\;shock \end{array}\right.\text{,}$$

(20) $$S\left(\Delta \right)=\frac{\theta \pi \left(1-\theta \right)\left(v^{H}-v^{L}\right)}{\left[\theta \pi +0.5\left(1-\pi +\Delta \right)\right]\left[\left(1-\theta \right)\pi +0.5\left(1-\pi -\Delta \right)\right]}\;\text{.}$$

Then based on Eq. (20) $S_{-}>S_{+}$ if and only if S(|Δ|)>S(−|Δ|). As the numerator takes on a constant value in the function, we focus on the denominator value f(Δ). Then, $S_{-}>S_{+}$ if and only if f(|Δ|)<f(−|Δ|), where

(21) f(Δ)=[θπ+0.5(1−π+Δ)][(1−θ)π+0.5(1−π−Δ)]

is a concave, second order polynomial function of Δ in Eq. (21). If and only if the maximum place of this function is reached in its negative domain, then f(|Δ|)<f(−|Δ|) is always true. Therefore, it is enough to test whether, as in Eq. (22),

(22) $$\underset{\Delta }{\mathrm{argmax}}f\left(\Delta \right)<0\text{.}$$

According to the first order condition

0.5[(1−θ)π+0.5(1−π−Δ)]−0.5[θπ+0.5(1−π+Δ)]=0,

(23) Δ=(1−2θ)π.

Hence, based on Eq. (23), if and only if θ>0.5, then $\underset{\Delta }{\mathrm{argmax}}f\left(\Delta \right)<0$, f(|Δ|)<f(−|Δ|), S(|Δ|)>S(−|Δ|) and $S_{-}>S_{+}$. In other words, if the probability of a subsequent higher value is greater than that of a lower value, then spread is greater subsequent to a negative shock than a positive shock. Using the same logic the above results apply to the sensitivity of spreads with respect to the ratio of heuristic-driven traders as well (see Appendix C) - higher participation of such class of investors results in larger (initially smaller) spreads subsequent to negative (positive) shocks in investors’ portfolios.

**Fig. 1:**
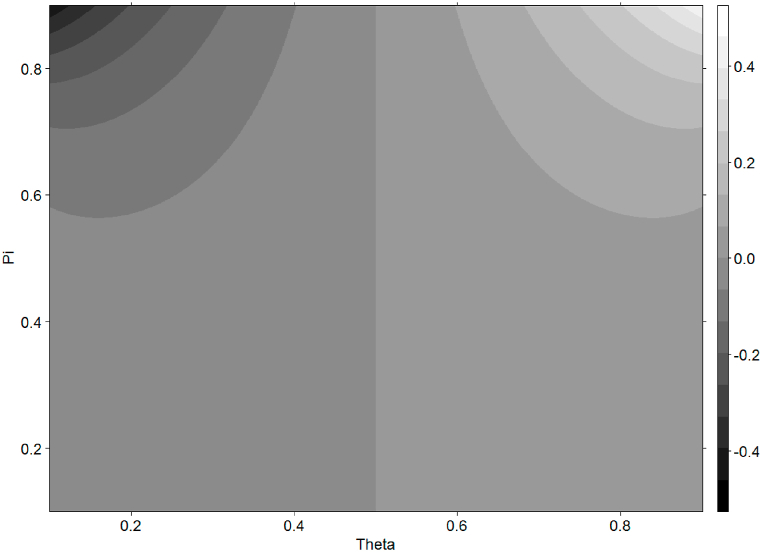
The formation of spread if heuristic-driven trading is present Notes: The figure represents the spread values according to the color bar to the right. The axes, Theta and Pi stand for the probability of high value and the probability of informed traders. The difference between high and low values, $v^{H}-v^{L}$ is set to one. The probability of heuristic-driven traders is defined by δ=0.1.

The economic intuition behind an average θ>0.5 is simple, as the growth of value is one of the basic assumptions in analyzing capital markets. This greater probability of a higher value is confirmed by empirical studies as well, although the authors apply a slightly different methodology [46]: Easley et al. (2002) and [47] Brennan et al. (2015) measure the probability of an increase in the value to be $P\left(v|v=v^{H}\right)=0.67$ and 0.614. The aforementioned pattern is represented in Fig. 1, where the spread (defined as in Eq. (20)) is shown as a function of θ and π: the calculation is made for the constraints of θ, π∈[0.1,0.9], δ=0.1, $v^{H}-v^{L}=1$. Irrespective of probability of informed trading, π (shown in the vertical axis), the difference of spread is clearly positive for a probability of higher value (shown in the horizontal axis), θ>0.5 and negative for θ<0.5 if the probability of heuristic-driven investors is positive.

In conclusion, we argue that, on average, the spread increases subsequent to losses and initially decreases subsequent to gains. This asymmetric change is a polynomial but convex function of the probability of such heuristic-driven traders (i.e. the larger the shocks, the proportionally more the participation of such traders in the market, and hence spreads follow accordingly). Given our assumption of investors holding a portfolio correlated with the market, the above finding applies to all risky and tradable assets (i.e. demand or supply changes as a time-series function of market shocks), although it may be stronger for assets where the average participation of heuristic-driven trading is more prominent. Moreover, considering that continuous market orders at the ask and bid prices define the standard deviation of price changes (see list of related literature below), our explanation clearly implies that previous positive (negative) shocks decrease (increase) both the spread and the volatility accordingly.

Related literature provides support to our aforementioned reasoning. According to Ref. [48] Ormos and Timotity (2016a), there is a clear and robust contrarian pattern due to the presence of such investors in the market microstructure, and expectations seem to be anchored to past prices and returns ([49] Ormos and Timotity, 2016b) [50]. Park and Sabourian (2011) analyze a similar setting based on the Glosten-Milgrom model, and find that people act as contrarian if their information leads them to concentrate on middle values [51]. Kaniel et al. (2008) [52], Choe et al. (1999) [53,54], Grinblatt and Keloharju (2000, 2001) [55], Richards (2005), and [56] Bloomfield et al. (2009a) also confirm the existence of such contrarian traders. Moreover, according to Ref. [57] Lof (2014), the introduction of contrarian trading in asset pricing models dramatically increases the predictive power of those models. 10.13039/100014337Furthermore, our former mental framing based explanation for the contrarian activity is supported by Ref. [58] Yao and Li (2013), who argue that prospect theory investors can behave as contrarian noise traders in a market, while [59] Kadous et al. (2014) finds that investors act as contrarians if, and only if, they have in the past held the particular asset that they buy in the subsequent period; this provides evidence that mental framing could indeed be responsible for negative feedback trading, rather than an alternative, exogenous factor. Related literature also indicated some evidence for the concurrent spike of spreads and heuristic-driven (contrarian) trading activity around significant negative shocks ([48] Ormos and Timotity 2016a). The final block of our framework, the positive effect of spreads on price volatility is well documented by Ref. [60] Phan et al. (2015) [61], Wang et al. (2015) [62], Cristelli (2013) [63], Hussain (2011) [64], Wang and Yau (2000), and [65] Wyart et al. (2008).

## 3 Empirical results

In this section we present the empirical analyses in two different ways: first, we test investors' dynamic behavior on a large sample containing individual trading data; second, an empirical parameter estimation of our volatility model is provided using the CRSP database consisting of the daily log-returns of the Standard and Poor's 500 index constituents listed on 10 September 2014. The analyzed period covers 21 years from September 10, 1993 to September 10, 2014.

### 3.1 Patterns in intertemporal choice

Losses and gains induce risk-seeking and more risk-averse behavior respectively. We argue that this behavior is a response to loss-aversion in a dynamic context, that is, investors are reluctant to realize losses (either physically or mentally) and try to break even ([23] Odean, 1998) in order to obtain their initial benchmark on average. According to equilibrium asset pricing, a higher required return, which compensates for the previous loss, is only reachable by investing in assets with increased risk; therefore, when combined with the change in risk attitude, losses increase the volatility of returns in the subsequent period. Gains follow the opposite pattern: investors are afraid of losing their previous wealth, and hence invest in less risky portfolios since the initial benchmark level is still reachable with the latter.

The data and methodology of our analysis is as follows: the sample we use is similar to that of [8] Barber and Odean (2000), consisting of the transactions and descriptive data of 158,006 accounts at a large discount brokerage firm from January 1991 to December 1996. In this paper we aim at defining the change in the riskiness (as measured by volatility) of investors’ portfolio; therefore, only common stock investments are considered, since a meaningful amount of historical returns and realized volatility can only be calculated for these assets. Nevertheless, findings in this reduced sub-sample should be representative for the whole sample, as the former account for 64 % of the latter, as measured by the number of observations. Altogether, the dataset containing at least one common stock transaction in the period includes 104,225 accounts, which can be further decomposed based on the type of the account, in which we apply cash, IRA, and margin accounts as control variables, and the equity held by the related household at the end of the period. We present the descriptive statistics of these sub-samples in Table 1.

**Table 1:** Descriptive statistics of the sample.

	All accounts	Cash accounts	IRA accounts	Margin accounts
**Num. of accounts**	104,225	22,995	37,155	10,328
**Mean equity**	68,293	39,859	48,988	47,953
**Median equity**	18,288	8419	21,549	4426
**St. dev. of equity**	300,450	129,257	129,017	247,607
**Num. of trades**	1,969,747	260,039	486,889	255,759
**Mean number of trades**	19	11	13	25

Notes: This table shows the descriptive statistics of the trading accounts included in our dataset.

In return calculations we use different types of mental frames. First, we assume that when selling occurs, the profit is measured as the sale price relative to the pre-transaction average purchase price of an asset. However, as the long position in an asset may include numerous buy transactions before sale of the stock, we argue that if the representativity or anchoring heuristics are responsible for the change in the risk attitude, the most recent information (i.e. the price of the last buy transaction) is the main factor in utility perception. Having calculated the gain or loss, the asset into which the realized money flows in the subsequent buy transaction is defined. Related to both the bought and sold assets the variance and standard deviation of daily returns in the preceding year are calculated. Finally, based on the aforementioned parameters, regressions are estimated to analyze whether the risk of the targeted asset is driven by the previous outcome.

The first regression (first 2 columns in Table 2) applies the simple OLS estimation of the variance of the targeted asset, including the profit (the return based on the average buy price) of the previous transaction as the independent variable, that is

(24) $${\sigma_{b,i}}^{2}=\hat{\alpha }+\hat{\beta_{1}}{\bar{r}}_{s,i}+e_{i}\text{,}$$

where ${\sigma_{b,i}}^{2}$ and ${\bar{r}}_{s,i,}$ stand for the variance of the asset in the subsequent buy transaction and the average return of the realized sell transaction of each *i* trade pair (i.e. a sell transaction and the first subsequent purchase) respectively in Eq. (24).

**Table 2:** Regression results.

Panel A											
		Subsequent σ^2^				Subsequent σ^2^				Subsequent σ^2^	
		Coef		p-value		Coef		p-value		Coef	p-value
**(Intercept)**		2.32E-03		0.0000		2.32E-03		0.0000		2.22E-03	0.0000
**Average return**		−8.60E-05		0.0010		–		–		–	–
**Return on the last trade**		–		–		−9.47E-05		0.0005		−1.09E-05	0.6885
**Previous variance**		–		–		–		–		4.94E-02	0.0000
**Adjusted R-squared**		0.0000		–		0.0000		–		0.0026	–
						Panel B					
	Subsequent σ^2^				Subsequent σ^2^				Subsequent σ		
	Coef		p-value		Coef		p-value		Coef		p-value
**(Intercept)**	2.21E-03		0.0000		2.17E-03		0.0000		3.00E-02		0.0000
**Previous variance**	2.21E-03		0.0000		4.69E-02		0.0000		–		–
**Expected return**					–		–		–		–
**Difference of last return**	−1.63E-03		0.0003		–		–		–		–
**Positive diff. of last return**	–		–		4.72E-03		0.0000		6.65E-03		0.0071
**Negative diff. of last return**	–		–		−7.78E-03		0.0000		−1.48E-02		0.0000
**Previous volatility**	–		–		–		–		2.32E-01		0.0000
**Adjusted R-squared**	0.0027		–		0.0031		–		0.0386		–

Panel C								
	Subsequent σ		Subsequent σ		Subsequent σ if Equity ≥ Median		Subsequent σ if Equity < Median	
	Coef	p-value	Coef	p-value	Coef	p-value	Coef	p-value
**(Intercept)**	3.08E-02	0	3.08E-02	0.0000	3.09E-02	0.0000	3.16E-02	0.0000
**Difference of last return**	−4.11E-03	0.0135	–	–	–	–	–	–
**Positive diff. of last return**	–	–	5.53E-03	0.0251	7.49E-03	0.0429	2.43E-03	0.4627
**Negative diff. of last return**	–	–	−1.35E-02	0.0000	−9.93E-03	0.0098	−1.47E-02	0.0000
**Previous volatility**	2.30E-01	0	2.28E-01	0.0000	1.99E-01	0.0000	2.48E-01	0.0000
**Equity**	−2.06E-09	0	−2.06E-09	0.0000	−1.61E-09	0.0000	−5.31E-08	0.0000
**Cash dummy**	−5.33E-04	0.0008	−5.25E-04	0.0010	−5.60E-04	0.0213	−9.45E-04	0.0000
**IRA dummy**	−1.66E-03	0	−1.67E-03	0.0000	−3.16E-03	0.0000	−1.96E-04	0.2380
**Margin dummy**	1.50E-03	0	1.49E-03	0.0000	2.57E-03	0.0000	1.37E-04	0.4620
**Adjusted R-squared**	0.0419	–	0.0419	–	0.0348	–	0.0467	–

Panel D						
	Subsequent σ for cash account		Subsequent σ for IRA account		Subsequent σ for margin account	
	Coef	p-value	Coef	p-value	Coef	p-value
**(Intercept)**	3.00E-02	0.0000	2.84E-02	0.0000	3.06E-02	0.0000
**Positive diff. of last return**	5.11E-02	0.0000	7.26E-03	0.1573	−1.28E-02	0.0388
**Negative diff. of last return**	−9.60E-04	0.9195	−3.50E-03	0.4974	−1.77E-02	0.0009
**Previous volatility**	2.44E-01	0.0000	2.60E-01	0.0000	2.66E-01	0.0000
**Equity**	−6.65E-09	0.0000	−4.80E-09	0.0000	−9.63E-10	0.0000
**Adjusted R-squared**	0.0531	–	0.0565	–	0.0479	–

Notes: This table represents regression results for equations (24), (25), (26), (27), (28), (29) and their adjustments. The dependent variables are listed in the columns, the Coef columns represent the estimated coefficients for the parameters listed in the rows, whereas the p-value columns stand for the probability of an incorrect rejection of the zero null hypothesis.

In line with the above reasoning on anchoring or representativity, in the second regression we test whether the change in the definition of the return increases significance and goodness-of-fit. This estimation is shown in Eq. (25) where the previous profit $r_{s,i}$ is measured as the return on the price of the last transaction.

(25) $${\sigma_{b,i}}^{2}=\hat{\alpha }+\hat{\beta_{1}}r_{s,i}+e_{i}\text{,}$$

One may argue that the variance also correlates with the risk of the sold asset: an investor may have a preference for risky assets, which could lead to a biased estimation of $\hat{\beta_{1}}$ in the previous equation. Therefore, the third regression (Eq. (26)) includes ${\sigma_{s,i}}^{2}$ as the variance of the sold asset using the return on the last buy price respectively.

(26) $${\sigma_{b,i}}^{2}=\hat{\alpha }+\hat{\beta_{1}}r_{s,i}+\hat{\beta_{2}}{\sigma_{s,i}}^{2}+e_{i}\text{,}$$

According to equilibrium pricing, investors do expect a premium for risk; thus, their expected return is different from zero. When including non-zero expected return in the fourth regression, a new definition of return may provide a better fit to utility perception: here the perceived return is defined as the deviation from the historical (one year) expected return at the last buy transaction preceding the sell transaction of an asset. Thus, we assume that investors form their non-zero expectations at the time they invest into an asset, based on its performance in the past. Accordingly, as both the length of time between last buy and subsequent sell transactions and the risk of assets varies throughout the data, another adjustment is required: the expected return is not the same for each transaction; hence, we standardize the deviation from the expected return by dividing it by the number of days between the buy and sell transactions. Subsequent to this definition we use this daily average deviation from the expectation as an independent variable as in the following Eq. (27), where $t_{s}$ and $t_{pb}$ stand for the time when the sell and the previous buy transactions occurred, respectively:

(27) $${\sigma_{b,i}}^{2}=\hat{\alpha }+\hat{\beta_{1}}r_{std,s,i}+\hat{\beta_{2}}{\sigma_{s,i}}^{2}+e_{i}:r_{std,s,i}=\frac{r_{s,i}-E\left(r_{i}|t=t_{pb}\right)}{t_{s}-t_{pb}}$$

In order to be able to distinguish effects of previous gains from losses, we apply two separated variables in regression five as defined in Eq. (28):

(28) $${\sigma_{b,i}}^{2}=\hat{\alpha }+\hat{\beta_{1}}r_{-std,s,i}+\hat{\beta_{2}}r_{+std,s,i}+\hat{\beta_{3}}{\sigma_{s,i}}^{2}+e_{i}:r_{-std,s,i}=\min\left(r_{std,s,i},0\right),r_{+std,s,i}=\max\left(r_{std,s,i},0\right)$$

Having analyzed the effects of previous outcomes on risk attitude as measured by variance, we provide further tests that now include volatility. The importance of this additional analysis is already highlighted in section 2.2, where we mentioned that asset returns are driven by standard deviation rather than variance. Hence, in further regressions we apply volatility as the dependent variable. The sixth regression is the same as Eq. (28) except for the previously defined change in the definition of risk.

Our extensive dataset covers further parameters related to each trading account; in particular, the equity held at the end of the period and the type of the account is included as well. In further regressions we also apply these measures as control variables and investigate differences between the subgroups. These variable are reflected in the seventh regression that is defined as in Eq. (29), where $E_{i},D_{C,i},D_{I,i}$ and $D_{M,i}$ stand for the equity, the cash-type dummy, the IRA-type dummy, and the margin dummy of the account related to the *i*th transaction respectively.

(29) $$\sigma_{b,i}=\hat{\alpha }+\hat{\beta_{1}}r_{std,s,i}+\hat{\beta_{2}}\sigma_{s,i}+\hat{\beta_{3}}E_{i}+\hat{\beta_{4}}D_{C,i}+\hat{\beta_{5}}D_{I,i}+\hat{\beta_{6}}D_{M,i}+e_{i}\text{,}$$

Once again, similar to Eqs. (27), (28), in regression eight we modify Eq. (29) according to Eq. (28), that is, by separately estimating the coefficients of gains and losses. Then, in subsequent estimations we apply this latter frame in subgroup estimations to test whether the above pattern exists independently in all subclasses. Those trading cash, IRA or margin accounts and those with above or below median wealth may behave differently (see Ref. [66] Guiso's and Paiella's 2008 paper on wealth-dependence of risk-aversion); hence, in the ninth equation the effects for accounts with equity value above its median (i.e. the top 50 % of investors ranked by equity value) are estimated, whereas the tenth calculates coefficients for the bottom 50 %. In the last three regressions we estimate the effects for subgroups with a cash, IRA, and margin account types.

In Table 2 we present the empirical results of the estimations: results for groups of regressions one to three, four to six, seven to ten, and eleven to thirteen are shown in Panel A, B, C and D respectively.

Results of the first four regressions indicate that, regardless of the type of return, the aggregate effect of previous outcomes on risk attitude is significantly negative even if the previous variance is included, which supports our theory of intertemporal mental framing. Even though we find a minor increase in the significance by changing the reference point from the average return to the return relative to the price of the last buy transaction and relative to the historical expected return, the extremely low adjusted R-squared values indicate non-linear dynamics, or missing variables. Regression five, in which gains and losses are allowed to have distinct effects on the risk attitude, yields a possible reason for this finding: separating the previous outcomes by their sign improves the goodness-of-fit of the latter models.

Nevertheless, the real difference in the performance of the model is revealed by changing the risk measure to volatility: regression six shows that the adjusted R-squared value jumps, which supports our discussion on the linear relationship between standard deviation and expected return in Section 2.2.

Results of the volatility estimation of regression seven indicate four novel findings: first, the aggregate effect of previous outcomes is significantly negative again; second, equity has a negative effect on risk-appetite, indicating that, in line with related literature, investors holding larger amounts in capital assets invested into less risky portfolios; third, market participants with cash and retirement (IRA) accounts also avoid risk shown by their negative coefficient; fourth, margin account holders have a higher appetite for risk, as shown by the positive relationship between subsequent volatility and the margin dummy.

Altogether, regressions in Panel C all indicate a similar pattern as before: negative differences relative to the expected return have a significant and negative effect on the subsequent risk-appetite, whereas positive differences are either much less significant, or not significant at all. In particular, regressions nine and ten show that choices of high-income investors are just as sensitive to previous outcomes as low-income investors.

Regression results in Panel D show a somewhat mixed picture: although coefficients are not significant in all cases, the previous patterns apply to all subgroups, except for the coefficient of the positive previous return of margin account holders. In this group, both previous gains and losses are significantly negative, leading to lower and higher subsequent volatility respectively.

Altogether, we find similar results to the aggregated regression of Eq. (29) and its adjustment for separated gains and losses; although, for positive deviations from the expected return we find a statistically significant positive effect on subsequent volatility, we argue that the low p-values are due to the extremely high number of observations. Following [67] Lin et al. (2011), we provide a test for the economic significance and analyze the effect of increasing number of observations on the significance of the coefficients of the previous outcome. According to our theoretical explanation, positive deviations from the expected return are also negatively correlated with subsequent volatility; nevertheless, since volatility is non-negative, huge realized gains lead to exactly the same portfolio choice (i.e. the risk-free asset) as a gain just high enough to cover two subsequent periods of the required return. Therefore, positive returns higher than a relatively small level (at least twice of the expected return) cannot be ascribed to a linear relationship with volatility, but instead are driven by a random process. This leads to the fact that for a reasonable number of observations, where the case of “too big to fail” does not apply, p-values of the positive coefficient should not indicate a significant effect. The last three regressions in Panel C (regressions eight to ten), in which the p-value of the coefficient of previous gains is much higher than that of losses, suggest such relationship; however, for such high number of observations a tiny effect may prove to be significant. In Appendix B, we provide a detailed analysis of this latter effects.

Another way to handle the non-linearity problem of previous gains is to use a simple dummy variable for positive shocks. The intuition behind this idea is that if the expected return is very small compared to the positive shocks, then, shocks exceeding this expected return have a constant effect on volatility, since investors would not, and cannot, reduce their required return and portfolio volatility to values below zero: they hold assets providing at least the risk-free return with zero volatility. Therefore, there is a discontinuity in the model for gains, which can be handled with the use of a dummy variable. So, we compare the results of the aforementioned model by applying a dummy variable for gains, and the model assuming a linear relationship between previous gains and subsequent volatility. Table 3 represents our findings.

**Table 3:** Regression results of volatility dynamics.

	Subsequent σ			
	Coef	p-value	Coef	p-value
**(Intercept)**	3.08E-02	0.0000	3.11E-02	0.0000
**Positive diff. dummy**	–	–	−6.12E-04	0.0000
**Positive diff. of last return**	5.53E-03	0.0251	–	–
**Negative diff. of last return**	−1.35E-02	0.0000	−9.73E-03	0.0001
**Previous volatility**	2.28E-01	0.0000	2.29E-01	0.0000
**Equity**	−2.06E-09	0.0000	−2.07E-09	0.0000
**Cash dummy**	−5.25E-04	0.0010	−5.49E-04	0.0006
**IRA dummy**	−1.67E-03	0.0000	−1.67E-03	0.0000
**Margin dummy**	1.49E-03	0.0000	1.48E-03	0.0000
**Adjusted R-squared**	0.0419	–	0.0420	–

Notes: The table represents regression results for two regressions between previous outcomes and subsequent volatility. The dependent variable is listed in the columns, the Coef columns represent the estimated coefficients for the independent variables listed in the rows, whereas the p-value columns stand for the probability of an incorrect rejection of the zero null hypothesis.

The results indicate three important findings: first, by avoiding the discontinuity problem the regression model shows a negative relationship between previous gains and volatility instead of linearity; second, this relationship becomes much more significant than in the linear model, and therefore, all the variables have extremely low p-values; third, the adjusted R-squared also increases in the new model suggesting a better fit with the dummy variable. Hence, altogether the findings support the negative relationship proposed in our theoretical model.

All together we argue that our empirical results confirm the validity of the behavioral side of our explanation. The aggregate coefficient of previous outcomes is negative and significant everywhere, even in regressions where other control variables are included. In particular, it seems irrelevant whether we test the effect on low- or high-income investors – the pattern emerges for all of them. Thus, as a confirmation of the theoretical model, we find that previous outcomes do indeed affect asset allocation and, subsequent to losses and gains, yield a capital inflow into assets with higher and lower risk respectively. This finding is also confirmed in existing literature on mutual fund activity, in which a negative relationship was found between returns and subsequent cash inflows ([68] Warther, 1995 [69]; Goetzmann and Massa, 1999 [70]; Edelen and Warner, 1999), and between contemporaneous inflow of equity and bond funds ([71] Goetzmann et al., 2000).

### 3.2 Estimating the volatility model

Based on behavioral pattern presented above, in this section, our theoretical volatility model is tested from an empirical perspective. Here, the α and β parameters (as in Eq. (30) and in line with Section 2, 2.3.4), stand for the effect of intertemporal mental framing on subsequent volatility and the price of risk respectively. Although, in the current empirical investigation, *α* represents the aggregate effect on subsequent asset price volatility instead of the effect on an individual investor's portfolio risk, these coefficients are estimated according to Eq. (30), which latter is an alternative form of Eq. (2). Here, we note that this paper, has the limitation of not estimating each transmission step separately, hence the α estimated in the current setup is driven by a much wider range of factors than that defined for investors' portfolio volatility. Nevertheless, we argue that the two variables are similar in meaning (i.e. both definitions stand for the sensitivity of a specific volatility process to unexpected shocks); hence we indicate them with an identical symbol and estimate using the same equations.

The dataset we apply covers the return and volatility time series of the daily values of the CRSP value-weighted equity index using both weekly and monthly periods covering 21 years between September 10, 1993 and September 10, 2014. The current estimation aims to provide support to the aforementioned behavioural patterns emerging in the volatility process; therefore, the choice of frequency is based on the relevant literature using similar data (i.e. behavioural and pricing data combined), which mostly consists of monthly sampling ([8] Barber and Odean (2000)); nevertheless, to increase the number of observations and to allow for further robustness checks we have also run these results with weekly data. The periodic returns are defined as the sum of logarithmic daily returns. The volatility is calculated as the standard deviation of the daily returns during the given period; however, since this method gives the daily volatility, it is multiplied by the ratio of the standard deviation of weekly returns divided by the standard deviation of daily returns (the adjustment to weekly from daily sampling). The estimation is based on simulating an additional error term $e_{t}$ of Eq. (2), that is

(30) $$e_{t}=r_{t}-\left(r_{f,t}+\beta \sigma_{t-1}+\alpha \left(r_{t-1}-r_{f,t-1}-\beta \sigma_{t-1}\right)\right)\text{.}$$

where α∈[−1,0] and β∈[0,1]. Here, the error term is not homoscedastic, therefore, we define the standardized error *u*_*t*_ as per Eq. (31).

(31) $$u_{t}=\frac{e_{t}}{\sigma_{t}}\text{.}$$

First, the distribution of the error is estimated based on maximum likelihood. As Jarque-Bera tests clearly reject the null hypothesis of normality, the standardized $u_{t}$ is assumed to follow a scaled Student's-t distribution with $E\left(u_{t}\right)=0$. Therefore, the estimated parameters consist of the scaling *s* and degress of freedom *df.* Once again, we highlight here that the choice of the distribution is arbitrary in a sense that one may find other distributional assumptions more adequate; however, due to space limitation and in order to keep focus on the most relevant findings, we refrain from listing results with all kinds of return distributions. Nevertheless, we encourage our readers to test our methodology with alternative processes, which may be an excellent area of further research related to our current findings.

Due to non-linear likelihood optimization, the fitted distribution is particularly sensitive to the starting values of the degrees of freedom. Hence, we provide ten estimations for each α and β pair, in which the ten starting values of the degrees of freedom are numbers equally placed in a logarithmic scale between one and the number of observations. For example, our monthly analysis includes 1061 monthly returns; therefore, the applied starting values for the degrees of freedom are 1, 2, 5, 10, 22, 48, 104, 226, 489, and 1061. The starting value of the scaling parameter is always set to one. Out of the ten estimations, the one with the highest Kolmogorov-Smirnoff p-value (the best fit) is chosen.

Second, we apply a Kolmogorov-Smirnoff test for each *α* and *β* pair to measure the significance of the difference between the empirical and estimated distribution functions. These pairs include 10,201 sets consisting of the cross products of 101 equally placed *α* values between −1 and zero, and 101 equally placed *β* values between zero and 1. The higher the p-value, the better the fit; therefore, the $\left[\begin{array}{c} \alpha \\ \beta \end{array}\right]$ pair yielding the highest p-value indicates the best fit of a distribution conditional to $E\left(u_{t}\right)=0$. So, this latter pair provides the least significant error terms.

**Fig. 2:**
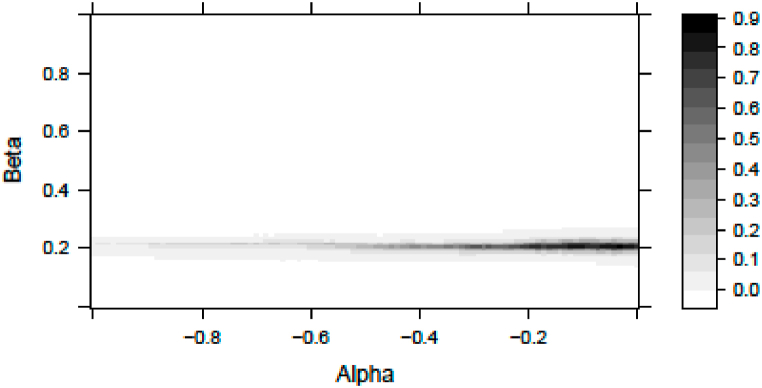
Kolmogorov-Smirnoff p-values in weekly analysis Notes: This figure represents the p-values of Kolmogorov-Smirnoff tests according to the color bar to the right. The tests apply the null hypothesis that the measured and the fitted samples come from similar distributions. We use maximum likelihood distribution fitting for the standardized error terms of Eq. (31) given the Alpha and Beta values plotted in the horizontal and vertical axes respectively. The statistics are valid for weekly sampled returns.

The numerical simulation for weekly data yields $\left[\begin{array}{c} \alpha \\ \beta \end{array}\right]=\left[\begin{array}{c} -0.03\\ 0.21 \end{array}\right]$. Here, the fitted distribution has a scaling *s =* 1.16 and a degree of freedom of *df = 6*. The probability that we incorrectly reject the null hypothesis of the Kolmogorov-Smirnoff test is 0.8535. In Fig. 2, we show the p-value (the goodness of fit) of the Kolmogorov-Smirnoff test as a function of *α* and *β*.

**Fig. 3:**
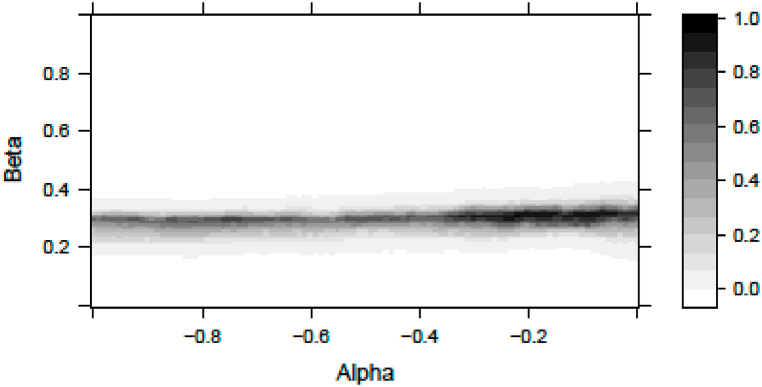
Kolmogorov-Smirnoff p-values in monthly analysis Notes: This figure represents the p-values of Kolmogorov-Smirnoff tests according to the color bar to the right. The tests apply the null hypothesis that the measured and the fitted samples come from similar distributions. We use maximum likelihood distribution fitting for the standardized error terms of Eq. (31) given the Alpha and Beta values plotted in the horizontal and vertical axes respectively. The statistics are valid for monthly sampled returns.

Monthly analysis indicates the best fit at the $\left[\begin{array}{c} \alpha \\ \beta \end{array}\right]=\left[\begin{array}{c} -0.22\\ 0.31 \end{array}\right]$ pair. The fitted distribution has a scaling *s =* 1.15 and a degree of freedom of *df = 225*. The Kolmogorov-Smirnoff p-value in this case test is 0.9481. Fig. 3 represents the p-value (the goodness of fit) of the Kolmogorov-Smirnoff test in the monthly results as a function of *α* and *β*.

Both results confirm our reasoning for the negative effect of previous shocks on subsequent volatility; moreover, the positive relationship between risk and required return remains intact. The particularly high *p-values* indicate that the error terms are well fitted using scaled *Student's-t* distributions.

## 4 Concluding remarks

We find that asymmetric and autoregressive volatility measured in previous empirical studies in asset pricing can be attributed to the intertemporal choice of investors, assuming that, at least in part, they are subject to mental framing. We show that, in contrast to some of the studies on asymmetric volatility, individuals tend to become less risk-averse (or risk-seeking until a given point), and more risk-averse subsequent to losses and gains respectively, which leads to the rejection of the volatility feedback and BHS explanations for asymmetric volatility. Furthermore, according to our results, the third main explanation (the leverage effect) does not hold either, as our analysis of the volatility process shows a significant volatility decreasing effect of both positive and negative asset-specific shocks when controlling for the market return.

However, our model predicts a negative relationship between market returns and market volatility, and is thus able to capture the dynamics of volatility measured empirically. Combining the linear relationship between risk and expected return with the aforementioned pattern in the intertemporal choice (i.e. the required return) yields an asymmetric autoregressive conditional heteroscedasticity model. We show that the discrete and continuous time alternatives of our main equation result in TGARCH and EGARCH models respectively – which, in particular, are measured to be two of best fitting frameworks in most empirical studies. Moreover, an empirical parameter estimation in discrete time indicates that the proposed model outperforms the simple random walk model, and supports the negative effect of previous outcomes.

Our findings are relevant for investors and industry participants for the following reasons. First, empirically validated models often indicate spurious correlations, even if the research is carried out in the most careful manner; in contrast, if a pattern is founded on behavioural traits that remain constant over decades or centuries it is much more likely to remain present in out-of-sample analysis as well (e.g. overreaction and mean-reversion of assets prices); hence, investors may trust more the asymmetric volatility process that has roots in behavioural theory. Second, if investors have access to information on market participants’ portfolio or trades, they may be able to estimate the future volatility of assets prices more robustly using the indirect method described in this paper.

Some potential avenues of further research: first, it would be interesting to see an experimental analysis that shows whether these patterns are found in a laboratory environment as well, if the focus is on the effect of breaking-even. Second, the influence of this behavior on asset liquidity and market microstructure could be analyzed in detail, including an empirical analysis of the probability estimation of heuristic-driven traders. Third, the application of the model in mathematical finance could reveal other interesting patterns; in particular, it could explain the skew of the implied volatility in the Black-Scholes option pricing model, and support asymmetric processes, such as in Ref. [72] Heston and Nandi (2000), or with an extension for higher lags, contribute to asymmetric Fractionally Integrated GARCH (FIGARCH) models ([37] Baillie et al., 1996). Fourth, the introduction of cognitive research, such as the neuroeconomic approach, could reveal additional underlying factors behind the behavioral patterns presented in this paper. Finally, as noted above, the current paper does not address the detailed empirical estimation of parameters at each step in the transmission of investor behavior into asset price volatility, hence further in-depth analyses may reveal important findings on how exactly other factors contribute to the components of this multi-step link.

## Data availability statement

The individual trading dataset was collected by Terrance Odean, he provided us for research purposes ([8] Barber and Odean, 2000), this data was available upon request. The historical daily log-returns of the Standard and Poor's 500 index constituents are available from Bloomberg or from CRSP. CRSP data requires subscription; however, partially it is available via Kenneth French Data Library.

## CRediT authorship contribution statement

**Mihály Ormos:** Conceptualization, Formal analysis, Funding acquisition, Investigation, Methodology, Project administration, Writing – original draft, Writing – review & editing. **Dusán Timotity:** Conceptualization, Data curation, Formal analysis, Investigation, Methodology, Software, Writing – original draft, Writing – review & editing.

## Declaration of competing interest

The authors declare that they have no known competing financial interests or personal relationships that could have appeared to influence the work reported in this paper.

## Figures and Tables

**Table A.1:** Weekly regression results

	1		2		3		4		6	
	Coef	p-value	Coef	p-value	Coef	p-value	Coef	p-value	Coef	p-value
**α**	0.0000	0.0991	0.0020	0.0000	0.0014	0.0000	0.0010	0.0000	–	–
**α+**	–	–	–	–	−0.0002	0.3489	–	–	–	–
**β1**	−0.0214	0.0000	0.0306	0.0000	0.1262	0.0337	0.0544	0.0000	0.0518	0.0000
**β2**	–	–	−0.1070	0.0000	−0.2301	0.0045	−0.1326	0.0000	−0.1339	0.0000
**β3**	–	–	–	–	–	–	−0.1252	0.0000	−0.0916	0.0000
**β4**	–	–	–	–	–	–	0.1621	0.0000	0.0281	0.0000

**Fig. A.1:**
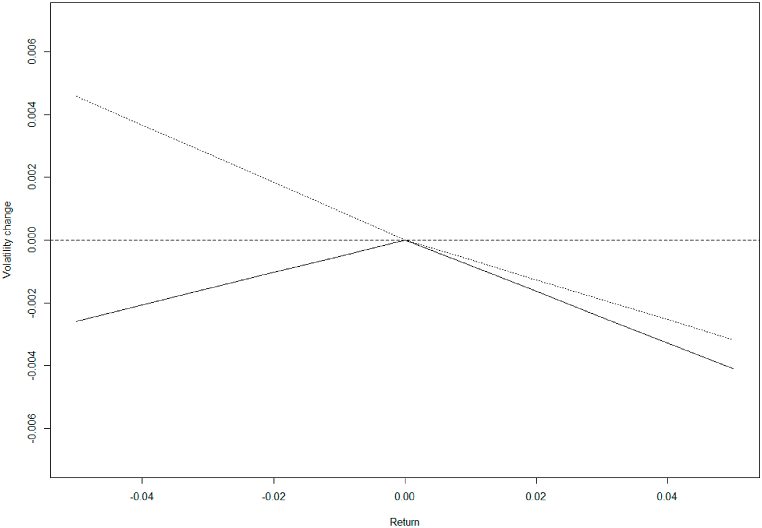
Effect of previous asset and market shocks

**Table A.2:** Monthly regression results

	1		6	
	Coef	p-value	Coef	p-value
**Α**	0.0000	0.2898	–	–
**β1**	−0.0080	0.0000	0.0169	0.0000
**β2**	–	–	−0.0423	0.0000
**β3**	–	–	−0.0317	0.0000
**β4**	–	–	0.0210	0.0000

**Table A3:** Robustness test results

	340 members		2007–2008		2009–2014	
	Coef	p-value	Coef	p-value	Coef	p-value
**α**	–	–	–	–	–	–
**β1**	0.0352	0	−0.0163	0	0.0019	0.105
**β2**	−0.1019	0	0.01	0.008	−0.0011	0.351
**β3**	−0.0776	0	−0.1058	0	−0.0494	0
**β4**	0.016	0	−0.0552	0.007	0.0021	0.558

**Fig. B.1:**
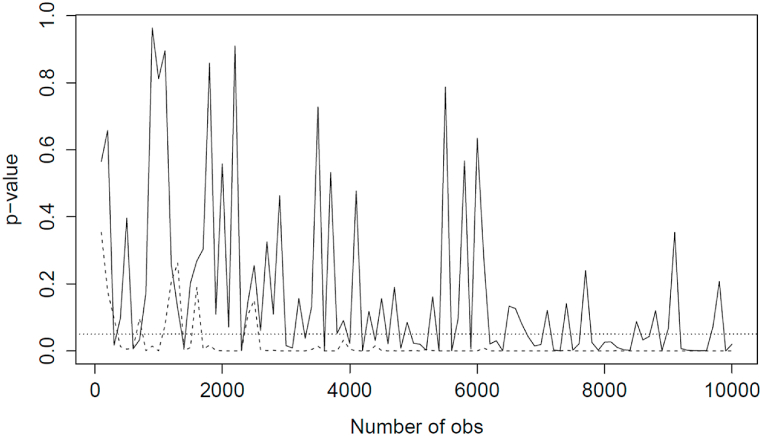
Coefficient p-values of previous outcomes as a function of the sample size

## Appendix A

In Appendix A, we provide empirical evidence that further develops the existing explanations of the asymmetric volatility effect. In theory, the volatility feedback would imply increased volatility following price shocks, regardless of their sign, while the leverage effect implies a negative correlation to the asset return. The data we use to test the hypotheses consists of the daily log-returns of the Standard and Poor's 500 index constituents on 10 September 2014 for a period period that covers 21 years, from September 10, 1993 to September 10, 2014. The purpose of this analysis is to test which asymmetry explanations hold in practice (in constrast to the mental framing we already established in Section 3.1).

### Methodology

First, the data is pooled together, therefore, the 501 (500 members and the index itself) time-series constitute the panel; then, we create two different panels by calculating the non-overlapping weekly and monthly returns by aggregating the daily log-returns. At the same time, a volatility panel is also generated by measuring the standard deviation of daily returns in each weekly and monthly period for each asset. As our aim is to measure the effect of previous shocks on the change of volatility the first regression is estimated as

(A.1) $$\Delta {vol}_{t,i}=\hat{\alpha }+\hat{\beta }r_{t-1,i}+e_{t,i}\text{,}$$

where $\Delta {vol}_{t,i}={vol}_{t,i}-{vol}_{t-1,i}$, $r_{t-1,i}$ and $e_{t,i}$ stand for the difference of volatility (i.e. standard deviation of daily returns in period *t*), the previous period return, and the error term of the *i-*th asset respectively. One might argue that positive and negative shocks have a different effect on the change of volatility, thus causing biased results. Therefore, we expand Eq. (A.1) with a variable $r_{t-1}^{+}$ that takes the value of the return if it is non-negative, and zero otherwise. The estimation yields the form of Eq. (A.2) as

(A.2) $$\Delta {vol}_{t,i}=\hat{\alpha }+\hat{\beta_{1}}r_{t-1,i}+\hat{\beta_{2}}r_{t-1,i}^{+}+e_{t,i}\text{.}$$

In order to test the discontinuity around zero return (in the ±0.05 quantile environment of the empirical distribution), a dummy for positive values, ${\hat{\alpha }}^{+}$ is tested as well; hence as in Eq. (A.3)

(A.3) $$\Delta {vol}_{t,i}=\hat{\alpha }+{\hat{\alpha }}^{+}+\hat{\beta_{1}}r_{t-1,i}+\hat{\beta_{2}}r_{t-1,i}^{+}+e_{t,i}\text{.}$$

We analyze the existence of volatility feedback by testing whether the coefficients of both previous gains and losses are significantly higher than zero. However, the interpretation of the leverage effect might still be misleading due to omitted factors. The latter explanation states that volatility is negatively correlated with previous asset returns; nevertheless, Eq. (A.3) may also include the contribution of other factors, such as the market return. Hence, the following estimation in Eq. (A.4) reveals the clean, decomposed effect of the previous market and asset returns on volatility:

(A.4) $$\Delta {vol}_{t,i}=\hat{\alpha }+\hat{\beta_{1}}r_{t-1,i}+\hat{\beta_{2}}r_{t-1,i}^{+}+\hat{\beta_{3}}r_{M,t-1}+\hat{\beta_{4}}r_{M,t-1}^{+}+e_{t,i}\text{,}$$

where $r_{M,t-1}$ and $r_{M,t-1}^{+}$ stand for the market return and the non-negative market return respectively. The inclusion of market returns in our setup is important even more so because $\hat{\beta_{3}}$ and $\hat{\beta_{4}}$ stand for the implications of our reasoning based on mental framing (i.e. it is the portfolio dynamics that cause the asymmetry in asset volatility, not individual asset price shocks). One may argue that due to data pooling, the average change of volatility ($\hat{\alpha }$) of separate assets differs causing biased estimation. The representation of Eq. (A.4) hence becomes Eq. (A.5)

(A.5) $$\Delta {vol}_{t,i}={\hat{\alpha }}_{i}+\hat{\beta_{1}}r_{t-1,i}+\hat{\beta_{2}}r_{t-1,i}^{+}+\hat{\beta_{3}}r_{M,t-1}+\hat{\beta_{4}}r_{M,t-1}^{+}+e_{t,i}\text{.}$$

Using the fixed effect approach, which filters out individual trends and demeans the variables with respect to time in this particular setting, yields Eq. (A.6) as

(A.6) $$\Delta {vol}_{t,i}-E\left[\Delta {vol}_{t,i}\right]=\hat{\beta_{1}}\left(r_{t-1}-E\left[r_{t-1}\right]\right)+\hat{\beta_{2}}\left(r_{t-1}^{+}-E\left[r_{t-1}^{+}\right]\right)++\hat{\beta_{3}}\left(r_{M,t-1}-E\left[r_{M,t-1}\right]\right)+\hat{\beta_{4}}\left(r_{M,t-1}^{+}-E\left[r_{M,t-1}^{+}\right]\right)+e_{t,i}\text{.}$$

### Empirical results

Table A1 summarizes the OLS estimation results of models from Eq. (A.1) to (A.6) (as indicated by numbers in the first row) using weekly statistics.

Including market return reveals an interesting pattern: changes of volatility are negatively correlated with previous market returns, whereas we find no correlation between previous asset return and volatility (thus, volatility decreases subsequent to both gains and losses). Contrary to the volatility feedback hypothesis, and McQueen and Vorkink's explanation, according to the negative aggregate effect (β1+β2) present in Eq. (A.1) to (A.6) for previous gains, volatility does indeed decrease. Furthermore, the positive coefficients in Eq. (A.2) to (A.6) of β1 reject the existence of the leverage effect, which would require a negative relationship between losses and the change of volatility. This finding is in line with the findings of [73] Hasanhodzic and Lo (2011): they underline that leverage effect is existent in the case of all-equity-financed companies as well, and therefore, reject the hypothesis.

In order to better represent the decomposed effect, Figure A1 shows Eq. (A.6) on the [−0.05,0.05] return interval where the solid and dotted lines represent the effect of previous asset and market return respectively. The dashed line stands for $\hat{\Delta {vol}_{t,i}}-E\left[\Delta {vol}_{t,i}\right]=0$.

We argue that the effects are driven by two distinct factors: on the one hand, the previous market return has indeed a negative correlation to the volatility of an asset. As we show below, this is due to the aggregate perception of utility caused by intertemporal mental framing; thus, investors turn to riskier assets providing a higher expected return in order to be able to break even and compensate for previous losses. On the other hand, the volatility-decreasing effect of previous shocks of a given asset is simply caused by the base effect: in case of massive shocks in the asset price the contemporaneous volatility rises and, since volatility follows a strong mean-reverting process, the unusually high or low level of volatility is followed by a decrease or increase respectively. This proposition can be recognized in the results that the lag-1 autocorrelation of the volatility difference of each asset is negative and is significant at 5 % in our sample: a rise in volatility is likely to be followed by a decrease.

In order to increase the robustness of these results a monthly test is also provided in Table A2, where we focus on regressions Eq. (A.1) and (A.6), omitting the transitory steps. The results are in line with the weekly analysis, and therefore, the pattern is robust regardless of the length of the period applied.

One may argue that these results are subject to selection bias (i.e. some members existing at the end of the examination period have no trading data at the beginning), and fluctuations toward the end of the period are over weighted. Therefore, another robustness test is presented in the first column of Table A3 including the 340 equities traded in both 1993 and 2014. Another argument against the latter regressions could be related to the 21-year period in use, therefore, in column 2 and 3, subperiods of 2007–2008, covering the main events of the recent financial crisis and that of 2009–2014 are estimated. These tests are provided only for weekly data in order to keep the number of observations sufficiently high and only Eq. (A.6) is estimated to compare the results.

These results confirm that market return is negatively correlated with the subsequent change in volatility, whereas the effect of the asset return either remains the same, or becomes minor in magnitude relative to that of the market return.

## Appendix B

In Figure B1 we present the coefficient p-values in the function of the number of observations, in which regression eight (as shown in Eq. B.1) is estimated with the following methodology: first, we draw 100 random sub-samples for each sample size, going from 100 to 10000; second, for each of these 100 random sub-samples regression eight is estimated; third, the p-value of the 100 the coefficients obtained for each sample size is defined.

(B.1) $$\sigma_{b,i}=\hat{\alpha }+\hat{\beta_{1}}r_{std,s,i}+\hat{\beta_{2}}\sigma_{s,i}+\hat{\beta_{3}}E_{i}+\hat{\beta_{4}}D_{C,i}+\hat{\beta_{5}}D_{I,i}+\hat{\beta_{6}}D_{M,i}+e_{i}\text{,}$$

We plot these p-values as a function of the sample size, where the continuous, dashed, and dotted lines stand for the p-values of gains, losses, and the 0.05 level respectively. One can clearly see that the coefficient of losses is always significant for sample sizes at least as high as 2500, whereas for gains, p-values are above 0.05 even for sample sizes around 10,000. We argue that this result supports our explanation of the economically insignificant effect of previous gains.

Notes: This figure represents the coefficient p-values of losses and gains in regression eight as the function of number of observations. We use random sampling in order to obtain lower number of observations. The solid, dashed and dotted line stand for the p-values of gains, losses and the 0.05 level respectively.

## Appendix C

Sensitivity of spreads to heuristic-driven trader participation in our model is captured by the derivatives of spreads (i.e. Eq. (C.1) and (C.2) instead of equations (18), (19)):

(C.1) $$\frac{\partial S_{-}}{\partial \delta }=\frac{\partial \frac{\theta \pi \left(1-\theta \right)\left(v^{H}-v^{L}\right)}{\left[\theta \pi +0.5\left(1-\pi +\delta \right)\right]\left[\left(1-\theta \right)\pi +0.5\left(1-\pi -\delta \right)\right]}}{\partial \delta }$$

(C.2) $$\frac{\partial S_{+}}{\partial \delta }=\frac{\partial \frac{\theta \pi \left(1-\theta \right)\left(v^{H}-v^{L}\right)}{\left[\theta \pi +0.5\left(1-\pi -\delta \right)\right]\left[\left(1-\theta \right)\pi +0.5\left(1-\pi +\delta \right)\right]}}{\partial \delta }$$

After a few modifications it becomes clear from the above that for negative shocks increasing the ratio of heuristic-driven investors always increases the spread if θ>0.5.

As for the spread following positive shocks, under similar conditions the derivative is initially negative for small positive shocks (i.e. low participation of heuristic traders) but become positive if δ>(θ−0.5)π. These “mixed” findings for the latter are in line with the milder aggregate effect of positive shocks to volatility.
